# Alfa-Lipoic Acid Controls Tumor Growth and Modulates Hepatic Redox State in Ehrlich-Ascites-Carcinoma-Bearing Mice

**DOI:** 10.1100/2012/509838

**Published:** 2012-05-01

**Authors:** M. AL Abdan

**Affiliations:** Zoology Department, Faculty of Science, Princess Nora Bint AbdulRahman University, Riyadh 11481, Saudi Arabia

## Abstract

The effect of oral supplementation of *α*-lipoic (LA) on growth of Ehrlich ascites carcinoma cells (EACs) and hepatic antioxidant state in mice was investigated. The results revealed that *α*-lipoic (LA) acid at 50 mg/kg body wt reduced the viability and volume of EAC cells and increased the survival of the treated animals. In addition, LA normalized oxidative stress in liver of mice-bearing EAC cells evidenced by increasing the levels of total thiols, glutathione, glutathione-S-transferase, superoxide dismutase, and catalyse. On the other hand, significant decreases in the levels of malondialdehyde and protein carbonyl were demonstrated in liver indicating controlled oxidative stress in these animals. As a consequence, LA regulated liver enzymes, alkaline phosphatase, aspartate aminotransferase, alanine aminotransferase, and gamma-glutamyl transferase. The data also indicated the efficiency of LA as cancer inhibitor and therapeutic influence. In conclusion, the present data suggest LA as a potential therapeutic complement in the treatment or prevention of different pathologies that may be related to an imbalance of the cellular oxidoreductive status associated with malignancy.

## 1. Introduction

Cancer is a major health problem in women and men all over the world. It is well known that there is an association between the incidence of cancer and diet, and nutritional factors can act as protective and therapeutic factors [1]. Initially, *α*-lipoic (LA) was obtained from livers and it has been found naturally in many plants and animals [2]. It is absorbed from the diet, biological membranes, and is then taken up by cells and tissues [3]. LA is easily absorbed and converted into the reduced form of dihydrolipoic acid in a variety of cellular tissues [4]. Both act as an antioxidant in different environments and mutually form a redox couple.

Besides acting as a potent antioxidant, LA increases and maintains levels of antioxidants such as ubiquinone, glutathione, and ascorbic acid [5, 6]. Furthermore, LA could be a potential therapeutic agent in the controlling different disorders that an imbalance of the cellular oxidoreductive status takes role [7, 8]. Oxidative damage has been implicated in cell injury, including possible participation in the formation and promotion of cancer.

Unlike normal cells, cancer cells can live in a redox environment where the elevated reactive oxygen species (ROS), which have been indicated as vital signalling molecules [9], contribute to promote cell proliferation and to suppress apoptosis. Previous studies indicated that intracellular redox balance is associated with cellular growth control. Moreover, it has been shown that dietary intake of antioxidants may be chemopreventive and may improve the efficacy of chemotherapy. The anticancer effects of LA were found in various cancer types such as ovarian epithelial cancer cells [10] and B16F10 murine melanoma cells [4]. However, the effect of lipoic acid on growth and proliferation EAC cells as mammary tumor is not studied. In addition, it is reported that LA increases the level of activity glutathione peroxidase in the body and reduces the oxidative stress [11] and therefore has a carcinostatic effect in cancer patients. But the anticancer effects of LA on breast cancer have not been fully understood. Ehrlich tumor is an experimental model for breast cancer. It grows in several strains of mice, in an ascitic form when inoculated in the peritoneal cavity and in the solid form when subcutaneously inoculated [12]. Ehrlich tumor is a species-specific, transplantable neoplasia from malignant epithelium that corresponds to mice's mammary adenocarcinoma [13]. Accordingly, the present study aimed to investigate the effect of LA on the EACC growth and its protective role on hepatic redox state in EACC-bearing mice.

## 2. Materials and Methods

### 2.1. Chemicals

All reagents were of the highest purity available. LA and all other chemicals were purchased from Sigma-Aldrich Company Ltd. (Gillingham, UK).

### 2.2. Cell Line

A line of Ehrlich carcinoma cell was used in this experiment. The tumor cells were maintained by serial interperitoneal transplantation of 1 × 10^6^ cells (in a volume of 0.1 mL saline) in adult female Swiss albino mice, weighing 20–25 g.

### 2.3. Determination of Viability *In Vitro*

Seven days after implantation, Ehrlich ascites carcinoma (EAC) cells were collected, diluted with cold saline, and divided into four suspensions. Three cell suspensions in phosphate-buffered saline (PBS) buffer were treated with LA at final concentration of (10, 20, 40 *μ*g/mL) for 30 min at 25°C. The fourth cell suspension was served as control. The viability of living cells using trypan Blue was checked according to the method of [14]. Results were given as the mean + SD of three independent experiments.

### 2.4. Animals and Experimental Treatment

Adult female Swiss albino mice weighing 18 to 20 gm were housed under the standard conditions in the animal house. They were fed standard pellet chow and allowed free access to water. All the experiments were carried out as per the guidelines of the institutional animal ethics committee. Ascitic carcinoma in mice was induced by injecting 1 × 10^6^ Ehrlich's ascitic carcinoma cells in the peritoneal cavity of mice as described [15]. LA was dissolved in saline solution and given orally at concentration of 50 mg/kg/day.

### 2.5. Determination of Mean Survival Time and Ascites Volume

Animals were inoculated with 1 × 10^6^ cells/mouse on day 0, and treatment with Lipoic acid started 24 hours after transplantation. The control group was treated with the same volume of saline solution. All treatments were given for 30 days. Mean survival time and ascites volume of each group, consisting of 20 mice, were recorded at 7, 15, and 30 days post implantation.

### 2.6. Experimental Design

The mice were divided into four groups. Group A: animals (*n* = 8) in this group received laboratory chow only and served as normal control. Group B: animals (*n* = 8) in this group were daily treated with LA orally. Group C: animals (*n* = 20) were inoculated with EAC. Group D: animals (*n* = 20) were implanted with EAC followed by daily oral administration of LA at concentration 50 mg/kg/day for 30 days. Ehrlich's ascitic carcinoma cells (10^6^ cells in 0.2 mL of PBS) were injected in the peritoneal cavity of the animals in groups C and D. The development of ascites was monitored by determining the Ehrlich's ascites carcinoma cell count in the peritoneal fluid, and the increase of cell count to 5 × 10^6^/mL was taken as the end point for the induction of the ascites in mice as an animal model system.

After completion of the experimental treatment of 15 days all animals were killed after 12 h of overnight fasting. Blood samples were collected into chilled nonheparinzed tubes and centrifuged at 3000 rpm for 10 min at 4°C. The sera were frozen at −20°C for biochemical analysis. Liver samples were isolated and homogenized in ice-cold (20 mM) phosphate-buffered saline (pH 7.4) for all the following biochemical assays.

### 2.7. Assay of Oxidative Stress

The levels of lipid peroxidation in liver were measured as thiobarbituric acid reactive substances (TBARSs), while the levels of protein carbonyl as indicator of protein oxidation were determined as describe [16, 17], respectively.

### 2.8. Antioxidants Assay

The activities of catalase (CAT), superoxide dismutase (SOD), and glutathione-S-transferase (GST) in the liver were assayed as mentioned before [18–20]. The levels of reduced glutathione (GSH) and total thiols (T-SH) were assayed as described earlier [21, 22], respectively. All protein concentrations were determined as described before [23].

### 2.9. Statistical Analysis

Results were expressed as mean ± SE. Differences between groups were assessed by ANOVA using the SPSS 13 software package for Windows. Post-hoc testing was performed for intergroup comparisons using the least significance difference (Tukey) test, significance at *P* values <0.05.

## 3. Results

The effect of different concentrations of LA (10, 20, 40 *μ*g) on the viability of EAC cells *in vitro *is displayed in Table 1. There was gradual decrease in the viability with increasing LA concentrations in a dose-dependent type (*r* ^2^ = 0.97 ± 0.09).

In Table 2, the effect of daily treatment with LA on the survival time and the percentage of survivals was followed for 30 days. An increase in the survival time and percent of survivals of LA-treated mice previously implanted with EAC cells is evident. However, the remaining 10% of the animals in the group B that also received similar administration of EAC cells survived the assault for more than 30 days then died (Table 2).

It is observed that the volume of EAC cells was significantly decreased when implanted mice received daily LA treatment (Table 3). It is also recorded that LA did not completely prevent growth of EAC cells. It is also noted that no control implanted animals survived at 30 day as shown in Tables 2 and 3.

After 15 days of EAC inoculation, there were abnormal increases of TBARS and protein carbonyl levels in the liver of the EAC-bearing mice in comparison to control levels. LA treatment significantly suppressed the increase in LPO and protein carbonyl levels in the liver of LA + EAC-treated group compared with EAC implanted mice (Table 4). Nevertheless, oxidative stress markers were still higher than the normal control levels.

Changes in liver antioxidants after 15 days of EAC implantation were shown in Table 5. EAC-bearing mice demonstrated significant decreases in the concentrations of GSH and T-SH in comparison to control. Daily oral supplementation with LA of EAC-bearing mice significantly maintained the hepatic GSH concentrations near normal amounts. The activities of antioxidant enzymes in the liver of EAC-bearing mice were inhibited in comparison to control group. This is evidenced by increased percent inhibition of SOD activity and lower activities of CAT and GST in the liver homogenate after 14 days of EAC-implantation. All the EAC induced alterations in the antioxidant levels and enzymatic activities were significantly modulated by LA treatment. Moreover, LA-treated mice showed enhanced levels of GSH, T-SH and SOD, CAT, GST in compared with that of the normal control. The treatment with LA alone showed significant increase in the levels of hepatic T-SH, GSH, and GST in comparison with the normal control group.

The effect of LA on liver function parameters in EAC-bearing animals is presented in Table 6. Significant decreases were observed in ALP, AST, ALT, and GGT activities in the liver measured 15 days after EAC implantation. On the other hand, daily treatment with LA significantly ameliorated the inhibition in comparison with EAC bearing mice. However, these parameters were still higher than the normal control groups.

## 4. Discussion

Cancer is a pathological state involving uncontrolled proliferation of tumor cells and systemic injury. In the present study, we investigated the effects of LA on the proliferation of EAC cells and hepatic redox state as health indicator of EAC-bearing mice. Epidemiological investigation and laboratory studies have indicated that different compounds developed from natural sources exhibit antioxidant activity and play an important role in the treatment of many cancers [24]. Among these molecules, LA is of particular interest, since it has been shown to possess antitumor activity in different human cancer cells without affecting normal cells [11, 25].

The present study demonstrated that LA markedly reduced the growth of EAC cells *in vitro*. This is evident from decreased cell viability, decreased ascites volume, and increased survival time of mice treated daily with LA. In accordance with the present results, it has been shown that LA induces p27Kip1-dependent cell cycle arrest and apoptosis in MCF-7 human breast cancer cells [26]. It is also reported that the proliferation of ovarian epithelial cancer cells was significantly decreased in response to treatment with LA in a dose-dependant manner [10]. Additionally, it is reported that treatment of Jurkat and CCRF-CEM human T lymphoma leukaemic cells with LA led to the dose-dependent inhibition of DNA replication and cell proliferation [25]. Furthermore, it indicated that LA exerts an inhibitory effect on cell proliferation via epidermal growth factor receptors and Akt signal transduction and induces cancer cell apoptosis in MDA-MB-231 human breast cancer cells [27]. Taken together, these findings indicate that LA inhibits the proliferation of a wide variety of cancer cells and tumor promotion in addition to its potential use in the prevention of cancer.

EAC-bearing mice showed to be under higher oxidative stress than control animals indicated by elevated lipid and protein oxidation and reduced endogenous antioxidants in the liver. In correlation, it is reported a decrease in SOD activity in EAC-bearing mice which might be due to the loss of MnSOD activity in EAC cells and the loss of mitochondria [28], leading to a decrease in total SOD activity in the liver [29]. Reactive oxygen species (ROS) and other free radicals are believed to be important in tumor promotion and progression and considered the main cause of organ injury and systemic dysfunction [30, 31]. In the present study, LA was able to control the upsurge in the lipid and protein oxidation in liver via modulation of antioxidants levels in the liver of EAC-bearing mice. LA scavenges the singlet oxygen, and hydrogen peroxide, hydroxyl radicals and also chelates the ferrous ions involved in the production of hydroxyl radicals [3]. Excess ROS can damage hepatocytes and activate hepatic stellate cells [32, 33], which play a central role in liver damage and fibrosis [34]. We found that administration of LA to mice implanted with EAC inhibited the development of liver injury, as indicated by reductions of liver function enzymes.

If oxidative stress is involved in the origin of EAC-induced liver oxidative injury, then a successful antioxidant treatment should protect against that injury. LA promoted a significant balance of oxidative stress in LA + EAC-bearing mice as evidenced by decreases in the levels of TBARS and carbonyl protein associated with increases in GSH and total thiols concentrations, as well as the activities of GST, SOD, and CAT compared with EAC-implanted mice. It is suggested that LA might prevent the oxidation of free or protein-bound thiols, thereby protecting its antioxidant properties. This study confirms the results of other studies [35] and showed that LA regulates the GST activity in liver and corrects their deficient thiol status by increasing the levels of hepatic GSH and T-SH. The maintenance of the thiol groups of proteins is a protective mechanism against oxidative stress and therefore influences the function of some thiol-containing proteins [36]. Besides acting as a potent antioxidant, LA either increases or maintains levels of other low-molecular-weight antioxidants such as ubiquinone, glutathione, vitamin E, and ascorbic acid [6, 35]. LA can therefore function to reduce oxidative stress efficiently and protect cellular membranes which may block apoptosis and cell death [37].

The data on LA's protective effect in tissue is consistent with reports by different investigators that LA maintains liver function [38] however, the precise mechanism by which LA maintains cellular integrity is not well known. Since liver is the main center for glucose metabolism, it is likely that increase in metabolism of glucose by LA [39], and thus the lowering of the glucose concentration in the medium, would result in the reduction of ROS production, lipid peroxidation, and protein oxidation. Treatment with LA significantly improves glucose tolerance, insulin release, plasma NEFA, skeletal muscle mitochondrial biogenesis, and oxidative stress in rats [40]. Both lipid peroxidation and oxidation of proteins can cause reduction in the activities of enzymes and alterations in the structure and function of membranes due to thiols blockage [41, 42]. Meanwhile, LA stimulated expression of heat shock proteins in liver cells and decreased the oxidative stress marker 4-hydroxynonenal adducts in the liver and heart of rats with metabolic stress and diabetes [43, 44]. This may explain how LA protects liver structure and function. Therefore, LA is a potential therapeutic agent in the treatment or prevention of different pathologies that may be related to an imbalance of the cellular oxidoreductive status associated with malignant patients.

In conclusion, these results suggest that supplementation with LA that are thought to influence liver function may be an effective strategy for improving liver dysfunction in EAC-bearing mice in addition to its oncosatic effect.

## Figures and Tables

**Table 1:** Effect of different concentrations of *α*-lipoic acid (LA) on the percentage viability of Ehrlich ascites carcinoma (EAC) cells *in vitro*.

LA concentrations *μ*g/mL	0	10	20	40
% viability	100	90^a^	65^a^	30^a^

The experiments were performed in triplicates; data shown represent mean ± SD of three independent experiments.

^a^Significance (*P* < 0.05) as compared with untreated cells (0 *μ*g/mL).

**Table 2:** Effect of 50 mg/kg body wt oral administration of *α*-lipoic acid (LA) on percent of survival of mice from carcinogenic assault by Ehrlich ascites carcinoma (EAC) cells.

Group	% survival at 7 days	% survival at 15 days	% survival at 30 days
Control	100	100	100
LA	100	100	100
EAC	70	20^a^	0^a^
LA + EAC	100	70^ab^	10^ab^

^a^Significant at (*P* < 0.05) as compared with control group

^b^Significant at (*P* < 0.05) as compared with EAC group.

**Table 3:** Effect of 50 mg/kg body wt. *α*-lipoic (LA) (daily administration) on the volume (mL) of Ehrlich ascites carcinoma (EAC) cells in mice.

Group	7 days	15 days	30 days
EAC	6.1 ± 0.2	8.9 ± 0.4^a^	—
LA + EAC	1.3 ± 0.1^b^	3.1 ± 0.3^cb^	9.1 ± 0.5^cb^

Values expressed as mean ± SE

^a^Significant at (*P* < 0.05) as compared with control group

^b^Significant at (*P* < 0.05) as compared with EAC group at 7 days

^c^Significant at (*P* < 0.05) as compared with EAC group.

**Table 4:** Concentrations of thiobarbituric acid reactive substance, TBARS, and protein carbonyl PC in liver of different animal groups.

Parameters	Control	LA	EAC	EAC + LA
TBARS (nmol MDA/g protein)	65.6 ± 2.9	61.8 ± 2.5	123 ± 6.5^a^	93 ± 6.3^ab^
PC (*μ*mol DNPH/mg protein)	461 ± 26	470 ± 19	736 ± 27^a^	574 ± 22^ab^

Values expressed as mean ± SE

^a^Significant at (*P* < 0.05) as compared with control group

^b^Significant at (*P* < 0.05) as compared with EAC group.

**Table 5:** Activities of superoxide dismutase (SOD), catalase (CAT), and glutathione-S-transferase (GST) (as well as levels of glutathione, GSH, total thiols (T-SH)) in liver of different animal groups.

Parameters	Control	LA	EAC	EAC + LA
SOD (% inhibition)	8.5 ± 0.22	8.5 ± 0.16	77.6 ± 3.1^a^	29.7 ± 1.2^ab^
CAT (*μ*mol H_2_O_2_/sec/g protein)	6.3 ± 0.18	5.9 ± 0.34	2.4 ± 0.22^a^	5.1 ± 0.15^ab^
GST *μ*mol/min/g protein)	0.48 ± 0.01	0.59 ± 0.01^a^	0.19 ± 0.005^a^	0.42 ± 0.008^a^
GSH (mg GSH/g protein)	0.40 ± 0.005	0.51 ± 0.001^a^	0.11 ± 0.004^a^	0.27 ± 0.007^ab^
T-SH (*μ*g/mg protein)	18.4 ± 0.32	25.4 ± 0.26^a^	8.2 ± 0.5^a^	14.8 ± 0.3^ab^

Values expressed as mean ± SE

^a^Significant at (*P* < 0.05) as compared with control group

^b^Significant at (*P* < 0.05) as compared with EAC group.

**Table 6:** Activities of ALP (alkaline phosphatase), ALT (alanine aminotransferase), AST (aspartate aminotransferase) and GGT (gamma glutamyl transferase) in serum of different animal groups.

Parameters	Control	LA	EAC	EAC + LA
ALP K.A.U./100 mL	130.6 ± 14	125 ± 15	162.4 ± 11^a^	137 ± 10^ab^
ALT U/I	70.7 ± 3.5	71.8 ± 4.42	95.0 ± 4.3^a^	80.5 ± 4.1^ab^
AST U/I	54.5 ± 3.5	50.1 ± 3.2	65.1 ± 4.6^a^	56.2 ± 4.2^b^
GGT U/L	6.0 ± 0.09	6.1 ± 0.01	11.9 ± 0.9^a^	7.9 ± 0.08^ab^

Values expressed as mean ± SE

^a^Significant at (*P* < 0.05) as compared with control group

^b^Significant at (*P* < 0.05) as compared with EAC group.
